# Using random forest for reliable classification and cost-sensitive learning for medical diagnosis

**DOI:** 10.1186/1471-2105-10-S1-S22

**Published:** 2009-01-30

**Authors:** Fan Yang, Hua-zhen Wang, Hong Mi, Cheng-de Lin, Wei-wen Cai

**Affiliations:** 1 Automation Department, Xiamen University, Xiamen, 361005, P.R.C; 2 Department of Molecular and Human Genetics, Baylor College of Medicine, Houston, TX 77030, USA

## Abstract

**Background:**

Most machine-learning classifiers output label predictions for new instances without indicating how reliable the predictions are. The applicability of these classifiers is limited in critical domains where incorrect predictions have serious consequences, like medical diagnosis. Further, the default assumption of equal misclassification costs is most likely violated in medical diagnosis.

**Results:**

In this paper, we present a modified random forest classifier which is incorporated into the conformal predictor scheme. A conformal predictor is a transductive learning scheme, using Kolmogorov complexity to test the randomness of a particular sample with respect to the training sets. Our method show well-calibrated property that the performance can be set prior to classification and the accurate rate is exactly equal to the predefined confidence level. Further, to address the cost sensitive problem, we extend our method to a label-conditional predictor which takes into account different costs for misclassifications in different class and allows different confidence level to be specified for each class. Intensive experiments on benchmark datasets and real world applications show the resultant classifier is well-calibrated and able to control the specific risk of different class.

**Conclusion:**

The method of using RF outlier measure to design a nonconformity measure benefits the resultant predictor. Further, a label-conditional classifier is developed and turn to be an alternative approach to the cost sensitive learning problem that relies on label-wise predefined confidence level. The target of minimizing the risk of misclassification is achieved by specifying the different confidence level for different class.

## Background

Most machine-learning classifiers output predictions for new instances without indicating how reliable the predictions are. The application of these classifiers is limited in the domains where incorrect predictions have serious consequences. Medical practitioners need a reliable assessment of risk of error for individual cases [1]. Thus, given the prediction tailed with a corresponding confidence value, a system can decide whether it is safe to classify. The recently introduced Conformal Predictor (CP) [2-5] is a promising framework that produces prediction coupled with confidence estimation. The exploiters advanced a welcome preference for formal relationship among Kolmogorov complexity, universal Turing Machines and strict minimum message length (MML). They assumed the transductive prediction as a randomness test which returns nonconformity scores closely associated with the property of the iid distribution (identically and independent distribution) governing all of the examples. When classifying a new instance, CP assigns a p-value for each given artificial label to approximate the confidence level of prediction. CP is more than a reliable classifier of which the most novel and valuable feature is hedging prediction, i.e., the performance can be set prior to classification and the prediction is well-calibrated that the accurate rate is exactly equal to the predefined confidence level. It is impressive to see its superiority over the Bayesian approach which often relies on strong underlying assumptions. In this paper, we use a random forest outlier measure to design the nonconformity score and develop a modified random forest classifier.

Since reports from both academia and practice indicate that the default assumption of equal misclassification costs is most likely violated [6], the natural desiderata is extending CP to label-wise CP, which takes into account different costs for misclassification errors of different class and allows different confidence level to be specified for different classification of an instance. In this paper, we investigate the method to extend CP to label-conditional CP, which can solve the non-uniform costs of errors in classification.

Consider a classification problem E: The reality outputs examples Z^(n-1) ^= {(x_1_, y_1_),..., (x_n-1_, y_n-1_)} ∈ X × Y and an unlabeled test instance x_n_, where X denotes a measurable space of possible instances x_i_∈ X, i = 1, 2,... n - 1,...; Y denotes a measurable space of possible labels, y_i_∈ Y, i = 1,2,... n - 1,...; the example space is represented as Z = X × Y. We assume that each instance is generated by the same unknown probability distribution P over Z, which satisfies the exchangeability assumption.

### Conformal predictor (CP)

CP is designed to introduce confidence estimation to the machine learning algorithms. It generalizes its framework from the iid assumption to exchangeability which omits the information about examples order.

To construct a prediction set for an unlabeled instance x_n_, CP operates in a transductive manner and online setting. Each possible label is tried as a label for x_n_. In each try we form an artificial sequence (x_1_, y_1_),..., (x_n_, y), then we measure how likely it is that the resulting sequence is generated by the unknown distribution P and how nonconforming x_n _is with respect to other available examples.

Given the classification problem E, The function A_n_: Z^(n-1) ^× z_n _→ R is a nonconformity measure if, for any n ∈ N,

*α*_i _:= A_n_(z_i_, ⟦_1_,..., z_i-1_, z_i+1_,..., z_n_⟧)

i = 1,..., n - 1

(1) *α*_n _:= A_n_(z_n_, ⟦z_1_,..., z_n-1_⟧)

where [·] is a "bag" in which the elements are irrelevant according to their order. The symbol *α *denotes sample nonconformity score: the larger *α*_i _is, the stranger z_i _is corresponding to the distribution. In short, a nonconformity measure is characterized as a measurable kernel that maps Z to R while the value of *α*_i _is irrelevant with the order of z_i _in sequence.

For confidence level 1 - *ε *(*ε *is the significance level) and any n ∈ N, a conformal predictor is defined as:

(2) $$\Gamma^{\varepsilon }({\text{z}}_{1},...,{\text{z}}_{\text{n}-1},{\text{x}}_{\text{n}})=\left\{\text{y}\in \text{Y}:{\text{p}}_{\text{y}}=\frac{\left|\{\text{i}=1,...,\text{n}:\alpha_{\text{i}}\geq \alpha_{\text{n}}\}\right|}{\text{n}}>\varepsilon \right\}$$

A smoothed conformal predictor (smoothed CP) is defined as:

(3) $$\Gamma^{\varepsilon ,\tau_{\text{n}}}({\text{z}}_{1},...,{\text{z}}_{\text{n}-1},{\text{x}}_{\text{n}},\tau_{\text{n}})=\left\{\text{y}\in \text{Y}:{\text{p}}_{\text{y}}=\frac{\left|\{\text{i}=1,...,\text{n}:\alpha_{\text{i}}>\alpha_{\text{n}}\}|+\tau_{\text{n}}|\{\text{i}=1,...,\text{n}:\alpha_{1}=\alpha_{\text{n}}\}\right|}{\text{n}}>\varepsilon \right\}$$

where y is a possible label for x_n_; P_y _is called p value, which is the randomness level of z_n _= (x_n_, y) and also the confidence level of y being the true label; *τ*_n_, n ∈ N is a random variables that distributed uniformly in [0, 1]. Smoothed CP is a power version of CP, which benefits from p distributing uniformly in [0, 1].

Let Γ^*ε *^= {y ∈ Y: P_y _> *ε*}, and the true label of x_n _is denoted as y_n_,

If |Γ^*ε*^| = 1, we define it as a certain prediction.

If |Γ^*ε*^| > 1, it is an uncertain prediction.

If |Γ^*ε*^| = ∅ , it is an empty prediction.

If y_n _∈ Γ^*ε*^, we define it as a corrective prediction with confidence level 1 - *ε*. Otherwise, it is defined as an error.

When it comes to forced point prediction, CP selects the label with maximum p value as the prediction.

CP is originally proposed for online learning and Vovk [7] offered the theoretical proof that in the online setting in the long run the prediction set Γ contains the true label with probability 1 - *ε *and the rate of wrong prediction is bounded by *ε*. Especially the smoothed CP is exactly valid, i.e., the rate of wrong prediction is exactly equal to *ε*. This is summarized as the proposition of well-calibrated.

(4) $$\underset{n\to \infty }{lim}\frac{\sup(Err_{n}^{\varepsilon })}{n}=\varepsilon$$

with $Err_{n}^{\varepsilon }$ the number of error predictions at the confidence level 1 - *ε *(See [7] for detailed proof). Extensive experiments demonstrated that CP is also applicable to offline learning, which enlarge its applications.

Different nonconformity measures have been developed from existing algorithms, such as SVM, KNN and so on [9-11]. All the CPs have the calibration property, but the efficiency of CP largely depends on the designing of nonconformity measure [8]. Efficiency means the certain and empty prediction ratio in all predictions. Certain prediction is favourable because it is more informative than uncertain predictions. CP is successfully employed to hedge these popular machine learning methods, and this paper shows that CP-RF is more efficient than others.

### Random forest (RF)

Breiman's random forest applies Bagging [12] and Randomization [13] technique to grow many classification trees with the largest extent possible without pruning. Random Forest is especially attractive in the following cases [14,15]:

(1) First, the real world data is noisy and contains many missing values, some of the attributes are categorical, or semi-continuous.

(2) Furthermore, there are needs to integrate different data sources which face the issue of weighting them.

(3) RF show high predictive accuracy and are applicable in high-dimensional problems with highly correlated features, especially in the situation which often occurs in bioinformatics, like medical diagnosis.

In this paper, the random forest outlier measure is used to design a nonconformity measure in order to incorporate random forest into the CP and label conditional CP scheme. Our method can be used in both online and offline settings.

### Cost-sensitive learning problem

In medical diagnosis, the default assumption of equal misclassification costs underlying machine learning techniques is most likely violated. A false negative prediction may have more serious consequences than a false positive prediction. To address this problem, cost-sensitive classification is developed, which considers the varying costs of different misclassification types [16]. Usually a cost matrix is defined or learned to reflect the penalty of classifying samples from one class as another. A cost-sensitive classification method takes a cost matrix into consideration during the model building process   [17]. However, how to get a proper cost matrix remains an open question [18]. The definition or learning of a cost matrix is quite subjective. In this paper, we extend our method to label conditional CP to address the cost sensitive problem, and the risk of misclassification of each class is well controlled.

## Results

### Experiments setup

The experiments are divided into two parts: First, to show the calibration property and efficiency of our method, we demonstrate our method CP-RF on 8 benchmark datasets and a real-world gene expression dataset. Second, to cope with the cost-sensitive problem, we extend CP-RF to label conditional CP-RF, and test its performance on two public application datasets.

### Part I Performance of CP-RF

We employ 8 UCI datasets [19], including satellite, isolet, soybean, and covertype, etc. Some details are included in Table 1, which contains information of the number of instances (*n*), number of class (*c*), number of attributes (*a*), and number of numeric (*num*) and nominal (*nom*).

**Table 1:** Datasets used in the experiments

Dataset	n	c	a	num	nom
liver	345	2	7	7	0

pima	768	2	8	8	0

sonar	208	2	60	60	0

house votes	435	2	16	0	16

satellite	6435	6	60	60	0

isolet	300	26	618	618	0

soybean	683	19	35	0	35

covertype	500	3	54	10	44

We perform CP-RF in a 10-fold cross validation in an online fashion and report the average performance and compare it with TCM-SVM and TCM-KNN. We use the following key indices at each predefined significance level: (1) Percentage of certain predictions. (2) Percentage of uncertain predictions. (3) Percentage of empty predictions. (4) Percentage of corrective predictions. These terms distinguish with traditional accuracy rate given by RF, SVM and other traditional classifiers.

**Figure 1:**
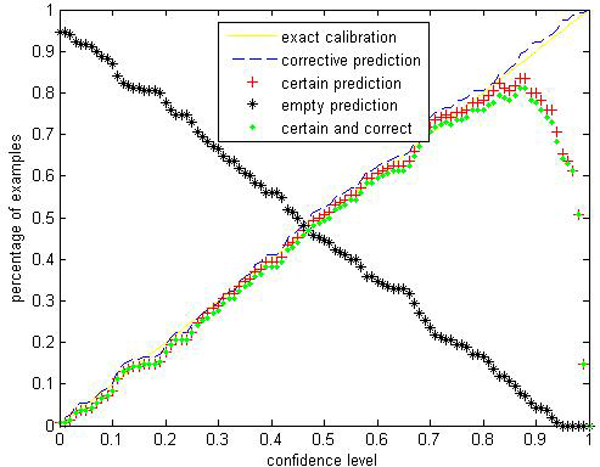
**Performance of CP-RF on pima**. We perform CP-RF by applying a ten-fold cross validation in online learning setting and report the average performance. To apperceive how accurate and effective the prediction region is, we use 4 evaluation indices at each predefined significance level: (1) Percentage of certain predictions. (2) Percentage of uncertain predictions with two or more labels which indicates that all these labels are likely to be correct. (3) Percentage of empty predictions. (4) Percentage of corrective predictions which give the proportion of test examples classified correctly. These terms are extended by conformal predictor and distinguish with traditional accuracy rate given by RF, SVM and other traditional classifiers.

**Figure 2:**
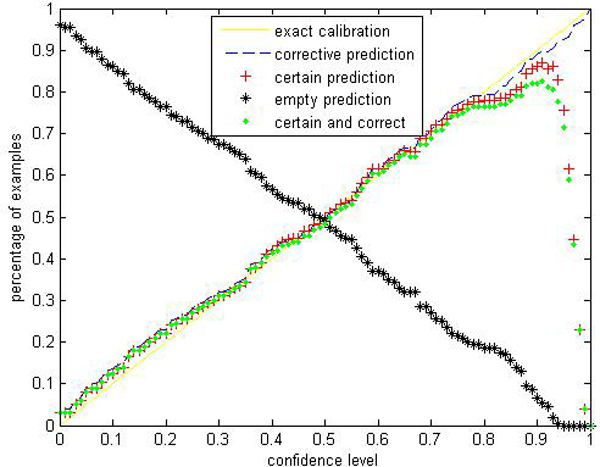
Performance of CP-RF on soybean.

**Figure 3:**
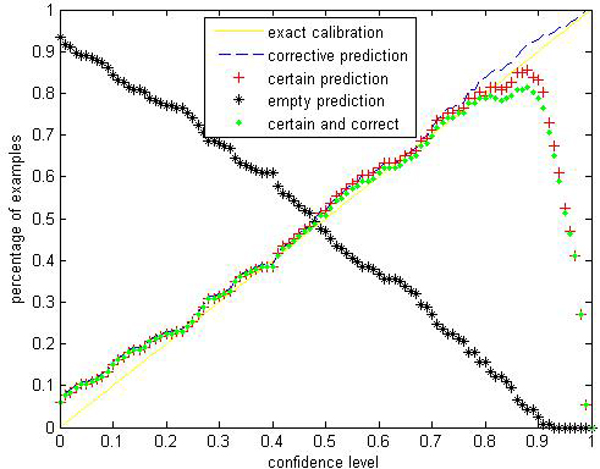
Performance of CP-RF on covertype.

**Figure 4:**
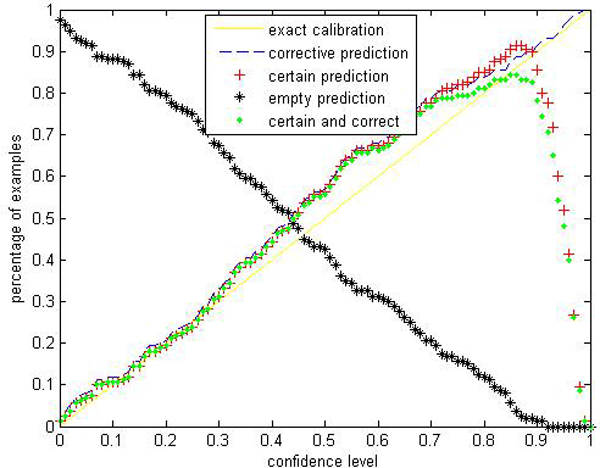
Performance of CP-RF on liver.

**Figure 5:**
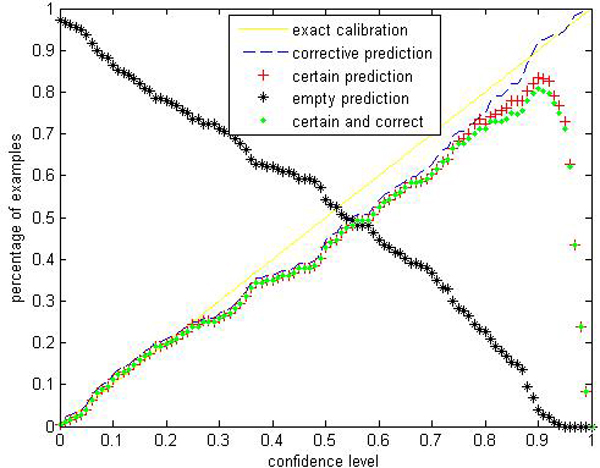
Performance of CP-RF on isolate.

**Figure 6:**
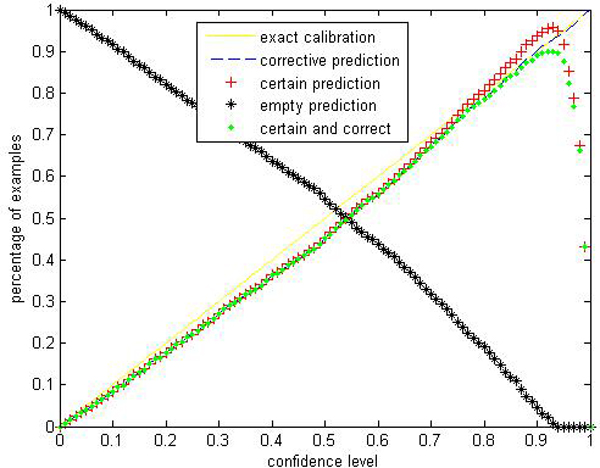
Performance of CP-RF on satellite.

**Figure 7:**
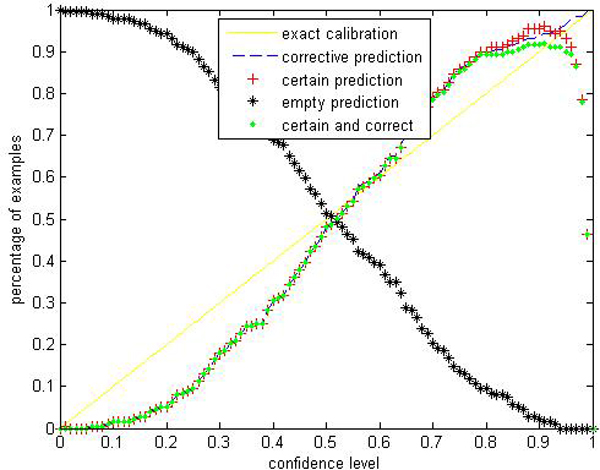
Performance of CP-RF on sonar.

**Figure 8:**
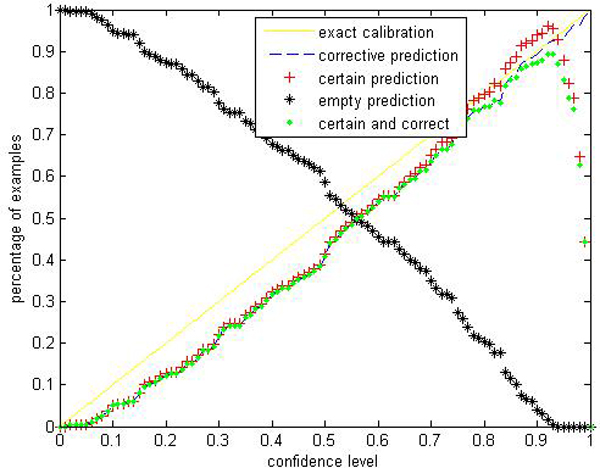
Performance of CP-RF on vote.

Given a significance level *ε*, the calibration and efficiency can be laid out. Let the number of trees (denoted as *ntrees*) equal to 1000 and the number of variables to split on at each node (denoted as *ntry*) be the default value $\sqrt{a}$ (*a *is the number of attributes). In Figures 1, 2, 3, 4, 5, 6, 7, 8, we demonstrate performance curves according with the significance level *ε *ranging from 0.01 to 1, and show the average experimental results on pima (continuous variables), soybean (categorical variables), covertype (mixed variables) and liver (poor data quality), etc.

Figures 1, 2, 3, 4, 5, 6, 7, 8 show that the empirical error line is well-calibrated with neglectable statistical fluctuations. It allows controlling the number of errors prior to classification. Percentages of corrective predictions with a predefined level of confidence illustrate the calibration of the new algorithm. Figures 1, 2, 3, 4, 5, 6, 7, 8 also show high accuracy with series of significance level and some interest points are extracted in Table 2 for impressive purpose.

**Table 2:** Corrective predictions at 5 confidence level

Confidence level	liver	pima	sonar	vote
0.95	94%	96%	96%	98%

0.90	80%	87%	93%	90%

0.85	78%	81%	82%	84%

0.80	66%	82%	76%	80%

Confidence level	satellite	isolet	soy-bean	cover-type

0.95	92%	94%	96%	96%

0.90	90%	88%	92%	82%

0.85	84%	85%	88%	78%

0.80	81%	79%	79%	76%

Table 2 demonstrates that CP-RF ensures relative high accuracy when controlling a low risk of error. It is important in many domains to measure the risk of misclassification, and if possible, to ensure low risk of error.

The percentage of certain predictions reflects the efficiency of prediction. Notice that the percentage of uncertain predictions monotonically decreases with higher significance levels. How fast this decline goes to zero depends on the performance of the classifier plugged into the CP framework. Figures 1, 2, 3, 4, 5, 6, 7, 8 show that CP-RF performs significantly well, and it is applicable at the significance levels of 0.20, 0.15, 0.10, and 0.05. For the convenience of comparison, we apply standard TCM-KNN and TCM-SVM algorithm provided by Gammerman and Vovk. The ratios of certain predictions on the 8 datasets are given for comparison in Table 3 and we can find that the efficiency of CP-RF is much better than the others, which indicates its superiority. Then we compare their performance in the occasion of forced point prediction in Table 4.

**Table 3:** Comparison of certain prediction

Dataset	Confidence level	CP-RF	TCM-KNN	TCM-SVM
Sonar	99%	53.15%	44.23%	23.07%
	
	95%	77.89%	73.07%	48.07%
	
	90%	86.74%	80.76%	71.15%

	Confidence level	CP-RF	TCM-KNN	TCM-SVM

Liver	99%	36.28%	17.56%	25.31%
	
	95%	67.21%	20.56%	31.45%
	
	90%	79.77%	36.08%	58.01%

	Confidence level	CP-RF	TCM-KNN	TCM-SVM

Pima	99%	42.36%	39.46%	24.05%
	
	95%	69.56%	44.56%	28.35%
	
	90%	74.71%	61.97%	45.12%

	Confidence level	CP-RF	TCM-KNN	TCM-SVM

Vote	99%	87.76%	83.49%	80.21%
	
	95%	92.87%	91.75%	90.72%
	
	90%	94.89%	92.56%	91.05%

	Confidence level	CP-RF	TCM-KNN	TCM-SVM

Satellite	99%	73.07%	64.47%	68.07%
	
	95%	87.92%	85.97%	88.41%
	
	90%	92.28%	90.72%	91.76%

	Confidence level	CP-RF	TCM-KNN	TCM-SVM

Isolet	99%	57.95%	54.87%	56.95%
	
	95%	74.72%	70.37%	72.07%
	
	90%	87.51%	81.75%	82.40%

	Confidence level	CP-RF	TCM-KNN	TCM-SVM

Soybean	99%	73.85%	54.57%	63.97%
	
	95%	79.32%	73.45%	78.83%
	
	90%	90.49%	80.29%	81.72%

	Confidence level	CP-RF	TCM-KNN	TCM-SVM

Covertype	99%	52.74%	47.82%	50.42%
	
	95%	68.73%	63.77%	65.07%
	
	90%	76.45%	72.16%	73.75%

**Table 4:** Comparisons of accuracy

Model	liver	pima	sonar	vote
CP-RF	66%	86%	84%	95%

TCM-KNN	61%	85%	83%	91%

TCM-SVM	51%	77%	96%	84%

level	satellite	isolet	soybean	covertype

CP-RF	84%	82%	93%	83%

TCM-KNN	82%	70%	89%	74%

TCM-SVM	74%	89%	77%	67%

It is clear that CP-RF performs well at most of the datasets, especially on the datasets with categorical and mixed variable. CP-RF especially outperforms TCM-KNN for high-dimension dataset (isolet), and outperforms TCM-SVM for noisy data (covertype).

To compare with the most popular machine learning methods, we consider Acute Lymphoblastic Leukemia (ALL) data which was previously analyzed with traditional machine learning methods. We choose an ALL dataset from [20] for comparison (see Table 5). There are 327 cases with attributes of 12558 genes. The data has been divided into six diagnostic groups and one that contains diagnostic samples that did not fit into any one of the above groups (labeled as "Others"). Each group of samples has been randomized into training and testing parts.

**Table 5:** The characteristic of ALL data

Group (Class)	Size of training set	Size of testing set
(1)BCR-ABL	9	6

(2)E2A-PBX1	18	9

(3)Hyperdiploid>50	42	22

(4)MLL	14	6

(5)T-ALL	28	15

(6)TEL-AML1	52	27

(7)Others	52	27

Total	215	112

We report results using CP-RF without discriminating gene selections, i.e. using all of the genes. In order to compare with traditional machine learning method, we apply CP-RF in an offline fashion, and use results of forced prediction. Table 6 demonstrates the detailed classification performance per class in confusion matrix, and it shows that CP-RF makes only 2 misclassifications. The comparison with a fine-tuned Support Vector Machine is laid out in Table 7.

**Table 6:** Confusion matrix of CP-RF

Real\Predicted	1	2	3	4	5	6	7
1	4	0	1	0	0	1	0

2	0	9	0	0	0	0	0

3	0	0	22	0	0	0	0

4	0	0	0	6	0	0	0

5	0	0	0	0	15	0	0

6	0	0	0	0	0	27	0

7	0	0	0	0	0	0	27

**Table 7:** Comparison of accuracy per class

Subgroups	CP-RF	SVM*
1	66.7%	100%

2	100%	100%

3	100%	99%

4	100%	97%

5	100%	100%

6	100%	96%

*The results of SVM are cited from [20]

Tables 6 and 7 show CP-RF outperforms SVM in subgroup 3, 4 and 6, and they are well-matched in subgroup 2 and 5. All of the two misclassifications happen in subgroup 1, because this subgroup only has six cases, the error rate seems very large.

Due to the low sample size, the reliability of classification is not guaranteed[21]. We show the distinct advantage of CP-RF with two measures, corrective predictions and certain predictions under 5 confidence levels in Table 8. The results show that our method is well-calibrated and make reliable predictions, even in an offline fashion.

**Table 8:** Corrective and certain prediction at 5 confidence levels

level	Corrective prediction	Certain prediction
99%	97.64%	100%

95%	93.24%	98.41%

90%	88.05%	98.32%

85%	83.90%	87.64%

80%	77.65%	82.94%

### Part II: Performance of label conditional CP-RF

In this part, we choose two multi-class and unbalanced real-world data sets as examples for cost sensitive learning. The objective is to control risk of misclassification within each class for different misclassifications may have different penalty in medical diagnostics. The first data set is the Thyroid disease records [22], and the problem is to determine whether a patient referred to the clinic is hypothyroid. Each record has 21 attributes in total (15 Boolean and 6 continuous) corresponding to various symptoms and measurements taken from each patient. The data set contains 7200 examples in total and is highly unbalanced in its representation of the 3 possible classes corresponding to diagnoses. Some details are included in Table 9, which contains information on the name, index and size of each class.

**Table 9:** Datasets used in the experiments

Name of class	Index	Size
1. Thyroid dataset		

primary hyperthyroid	1	166

compensated hyperthyroid	2	368

normal	3	6666

2. Chronic gastritis dataset		

incoordination between liver and stomach	1	240

dampness-heat of spleen and stomach	2	77

deficiency of spleen and stomach	3	151

blood stasis in stomach	4	84

yin deficiency of stomach	5	157

Another dataset is the Chronic Gastritis Dataset [23], which is a common disease of the digestive system with gastric inflammation being its notable features. Compare to Western medicine, Chinese medicine have many advantages in its treatment [24]. According to "*Diagnostic criteria for the diagnosis of chronic gastritis combining traditional and western medicine*" set by the Integrated Traditional and Western Medicine Digest Special Committee, Chronic Gastritis is divided into five subtypes (see table 9). In our application, we collected 709 cases from the digestion outpatient department of the Affiliated Shuguang Hospital during February and October, 2006. All cases are inspected by both gastroscopy and pathology. Each case is correlated with 55 kinds of symptoms listed in table 10.

**Table 10:** ID of the symptoms of chronic gastritis

ID	Symptom	ID	Symptom	ID	Symptom	ID	Symptom
1	distending pain	2	hunger pain	3	dull pain of stomach	4	stabbing pain of stomach

5	burning pain of stomach	6	abdominal distention	7	aggravated after eating	8	likeness of being warmed and pressed

9	aggravated in the night	10	distention and fullness	11	poor appetite	12	nausea

13	vomiting	14	vomiting	15	vomiting of water	16	belching

17	gastric upset	18	acid regurgitation	19	heartburn	20	blockage in deglutition

21	emaciation	22	dysphoria	23	sallow complexion	24	dim complexion

25	less lustrous complexion	26	cold limbs	27	dizziness	28	weakness

29	spontaneous sweating	30	night sweating	31	insomnia	32	dry mouth,

33	bitter taste in mouth	34	halitosis	35	loose stool	36	constipation

37	alternate dry and loose stool	38	hemafecia	39	yellowish urine	40	pale tongue

41	pink tongue	42	red tongue	43	purplish tongue	44	fissured tongue

45	teeth-print tongue	46	ecchymosis on tongue	47	thin-white fur	48	white and greasy fur

49	yellow and greasy fur	50	little fur	51	thready and unsmooth pulse	52	stringy and thready pulse

53	stringy and slippery pulse	54	deep and weak pulse	55	stringy and slippery pulse		

When constructing RF, we let the number of trees equal to 1000 and the number of variables to split on at each node be $\left\lfloor \sqrt{55}\right\rfloor$ (Parameter sensitivity analysis of CP-RF is laid out in the next section). For experiments on Thyroid disease dataset, the original dataset is randomly divided into a training set (3772 samples) and a test set (3428 samples). For Chronic Gastritis dataset, we perform our method in a 10-fold cross validation. Average performances are reported.

**Figure 9:**
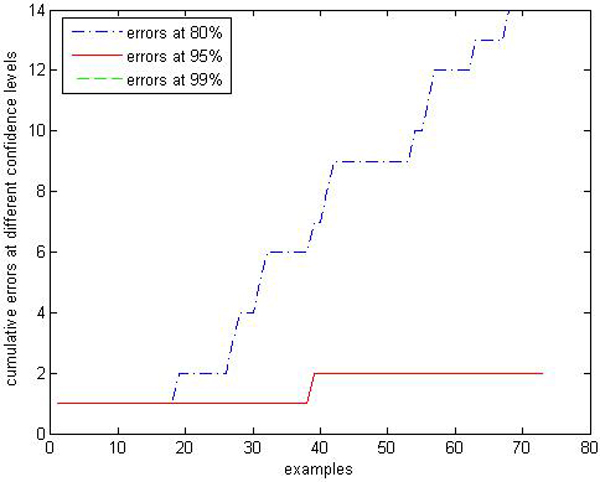
**Calibration Performance of CP-RF within class "primary hyperthyroid" on thyroid**. In this experiment, we apply CP-RF and label conditional CP-RF in an online learning fashion. The X axis represents the number of test samples within class "primary hyperthyroid", and Y axis represents the number of error predictions at 3 confidence levels (80%, 95%, and 99%).

**Figure 10:**
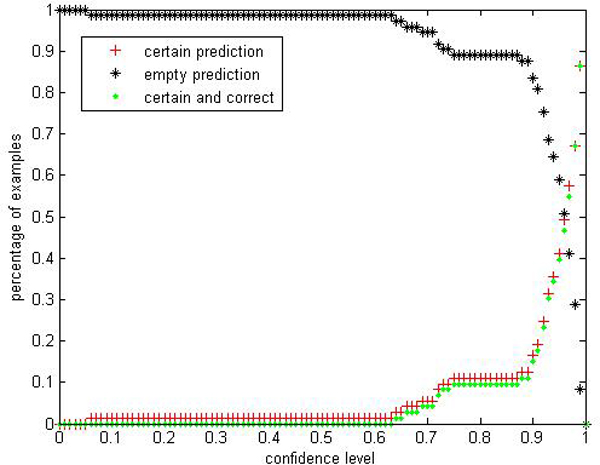
**Efficiency Performance of CP-RF within class "primary hyperthyroid" on thyroid**. To evaluate the performance of CP-RF within class "primary hyperthyroid", we show 3 indices in the experiment: certain prediction, empty prediction and certain&correct prediction.

**Figure 11:**
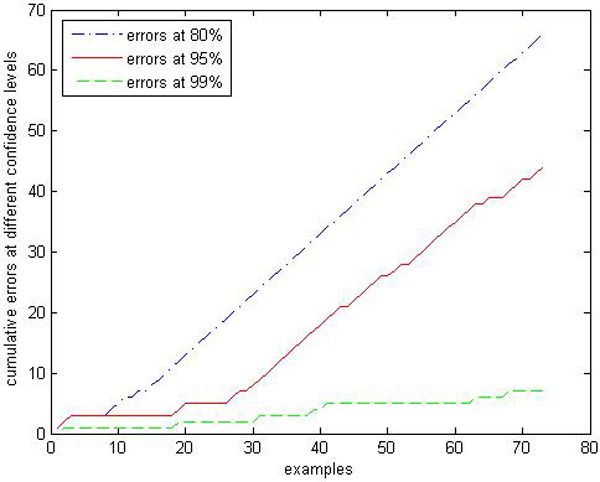
Calibration Performance of label conditional CP-RF within class "primary hyperthyroid" on thyroid.

**Figure 12:**
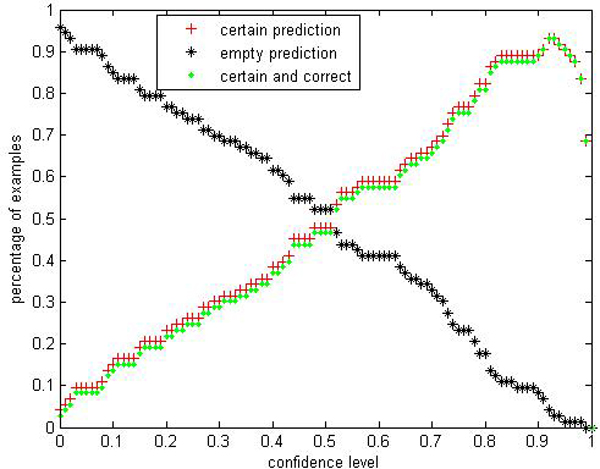
Efficiency Performance of label conditional CP-RF within class "primary hyperthyroid" on thyroid.

**Figure 13:**
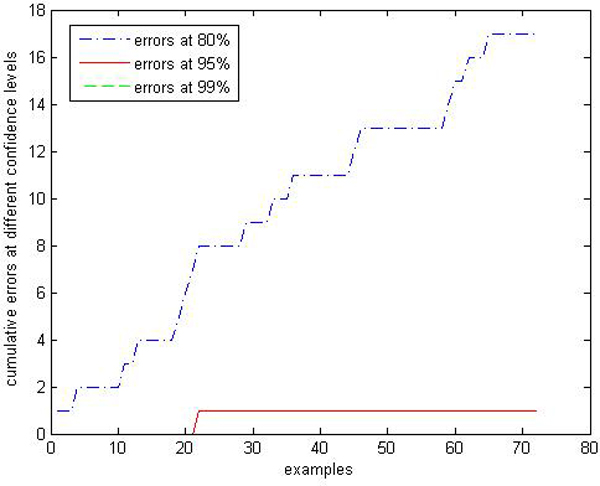
Calibration Performance of CP-RF within class "deficiency of spleen and stomach" on chronic gastritis.

**Figure 14:**
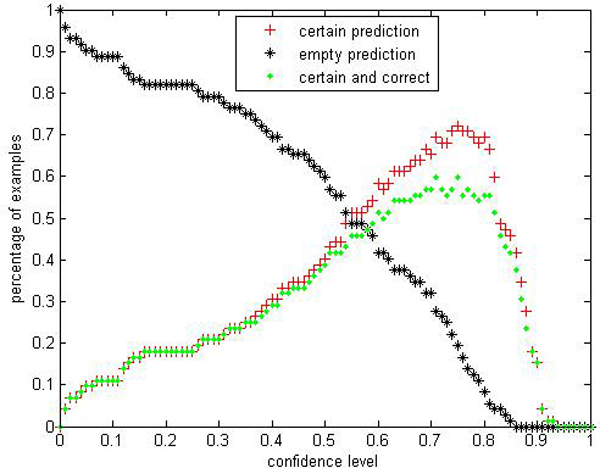
Efficiency Performance of CP-RF within class "deficiency of spleen and stomach" on chronic gastritis.

**Figure 15:**
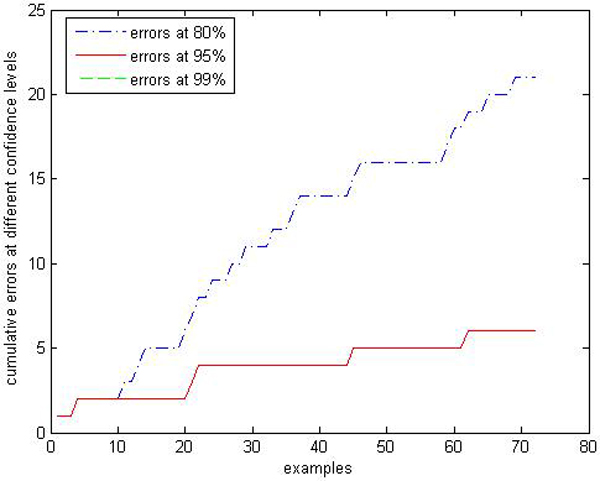
Calibration Performance of label conditional CP-RF within class "deficiency of spleen and stomach" on chronic gastritis.

**Figure 16:**
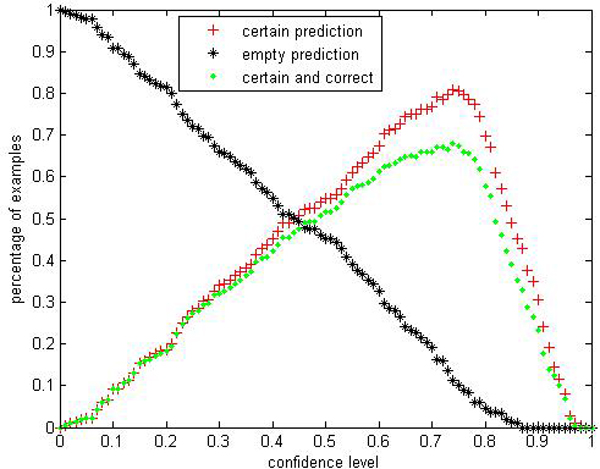
Efficiency Performance of label conditional CP-RF within class "deficiency of spleen and stomach" on chronic gastritis.

We are interested in performances comparison of the label conditional CP-RF with the CP-RF. Limited by space, we only show parts of results: Figures 9, 10, 11, 12, 13, 14, 15, 16 show experiment results on two classes with relatively small samples of Thyroid and Chronic Gastritis datasets. Figure 9 shows that the CP-RF is not well-calibrated at most of confidence levels within class "primary hyperthyroid" on Thyroid, and meanwhile Figure 10 shows that the efficiency of CP-RF is very low with confidences levels ranging from 0.01 to 1. In contrast, as is shown in Figure 11, label conditional CP-RF is well-calibrated up to neglectable statistical fluctuations and the empirical corrective prediction line can hardly be distinguished from the exact calibration line. Aside from the property of calibration, label conditional CP-RF show improvements on predictive efficiency in Figure 12, compared with CP-RF. These contrasts can also be observed in experiments on class "deficiency of spleen and stomach "of Chronic Gastritis datasets (See Figures 13, 14, 15, 16).

It is noticeable that the percentage of certain predictions and certain & correct ratios monotonically increase with significance levels. How fast the decline of uncertain prediction goes to zero also depends on the superiority of calculation of *p value*.

Some interest points are extracted in Tables 11 and 12. It demonstrates that label conditional CP-RF can be used to control the risk of misclassification within each class, so that it can be considered as an alternative approach for cost sensitive learning for unbalanced data.

**Table 11:** label conditional empirical corrective prediction at 5 confidence level within each class on thyroid data

class	99(%)	95(%)	90(%)	85(%)	80(%)
1	100	97.26	90.41	87.67	80.82

2	99.44	96.79	91.13	85.49	81.04

3	98.87	95.37	90.12	85.06	80.49

**Table 12:** label conditional empirical corrective prediction at 5 confidence level within each class on chronic gastritis data

class	99(%)	95(%)	90(%)	85(%)	80(%)
1	99.14	95.35	89.77	87.44	82.33

2	100	93.67	88.72	82.78	77.83

3	100	96.32	89.71	83.64	77.94

4	100	96.05	92.11	86.84	81.58

5	100	95.12	89.92	85.34	80.94

## Discussion

### Part I: CP-RF

#### Parameter sensitivity analysis

A common way to validate an approach is to ensure robustness, that is, the approach must produce consistent results independent of the initial parameter settings. Empirical studies show the parameters adjustments have great impacts on CPs. Normalization of examples affects TCM-KNN greatly. As for TCM-SVM, not only the normalization but the type and parameters of kernel functions are important. Thus, the empirical and non-theoretically alteration hints a potential instability.

To demonstrate the parameter insensitivity of CP-RF, we set up different parameters for CP-RF, with ntrees = 500,1000,5000 and ntry = 1,..., $\sqrt{\text{a}}$. Mean and standard deviation of forced accuracy on *sonar *are reported.

For TCM-KNN, We compare the fluctuation of forced accuracy with or without normalization; For TCM-SVM, the affection of different types of kernel are illustrated. The results on *sonar *are summed up in table 13. CP-RF shows a comparatively trivial fluctuation with the change of parameter settings. The advantage comes from the nature of RF and will benefit medical diagnosis.

**Table 13:** Comparison of Parameter Sensitivity

TCM-KNN	Without normalization	Attributes normalization	Examples normalization
accuracy	82.69%	88.46%	86.54%

TCM-SVM	Simple dot product	Radial basis function	Binomial coefficient polynomial

accuracy	63.46%	48.08%	96.15%

CP-RF	Mean	standard deviation	

accuracy	84.92%	3.52%	

#### Feature selection

The problem of feature selection is an open question in many applications. In our method, there is no feature selection. Take gene expression analysis for example, gene selection is a crucial study and remains unsolved. In Yeoh's study, gene expression profiling can accurately identify the known prognostically important leukemia subtypes, by the means of classification using SVM, KNN, and ANN when various selected genes were used. Unfortunately, classifications were performed following a process of discriminating gene selections by a correlation-based feature selection. This process is also labor intensive and requiring experiential knowledge. It is better that automated classification should be made with a level of confidence. Moreover, due to the low sample size, although their research has yielded high predictive accuracies that are comparable with or better than traditional clinical techniques, it remains uncertain how well the selected genes results will extrapolate to practice in the future [25]. CP-RF is especially suitable for this situation, without discriminating gene selections, i.e. using all of the genes, and this may meet the need of an automated classification. Moreover, no selection bias is introduced.

### Part II: Label conditional CP-RF

From experiments in Part I, we can see that though CP-RF is well calibrated globally, i.e. the error predictions equal to the predefined confidence level on the whole test data, it cannot guarantee the reliability of classification for each class especially for unbalanced datasets. Different from CP-RF, label conditional CP-RF is label-wise well calibrated while the former may not satisfy the calibration property in some classes. Because the latter uses only partial information from the whole data set, so the computational efficiency is better.

## Conclusion

Most of state-of-the-art machine learning algorithms cannot provide a reliable measure of their classifications and predictions. This paper addresses the importance of reliability and confidence for classification, and presents a novel method based on a combination of random forest, and conformal predictor. The new algorithm hedges the predictions of RF and gives a well-calibrated region prediction by using the proximity matrix generated with RF as a nonconformity measure of examples. For medical diagnosis, the most important advantage of CP-RF is its calibration: the risk of error can be well controlled. The new method takes advantage of RF and possesses a more precise and stable nonconformity measure. It can deal with redundant and noisy data with mixed types of variables, and is less sensitive to parameter settings. Furthermore, we extend CP-RF to a label conditional version, so that it can control the risk of prediction for each class independently rather than globally. This modified version can provide an alternative way for cost sensitive learning. Experiments on benchmark datasets and real world applications show the usability and superiority of our method.

## Methods

### CP-RF algorithm

Executed by transductive inference learning, CP is able to hedge the predictions of any popular machine learning method, which constructs a nonconformity measure for CPs [3,4]. It is a remarkable fact that error calibration is guaranteed regardless of the particular classifier plugged into CP and nonconformity measure constructed. However, the quality of region predictions and CP's efficiency accordingly, depends on the nonconformity measure. This issue has been discussed and several types of classifiers have been used, such as support vector machine, k-nearest neighbors, nearest centroid, kernel perceptron, naive Bayes and linear discriminant analysis [9-11]. The implementations of these methods are determined by the nature of these classifiers. So TCM-SVM and TCM-KP mainly consider binary classification tasks, TCM-KNN and TCM-KNC is the simplest mathematical realization, and TCM-NB and TCM-LDC is suitable for transductive regression. Indeed, the above methods have demonstrated their applicability and advantages over inductive learning, but there is still much infeasibility. For non-linear datasets, it is especially challenging to TCM-LDC. TCM-KNN and TCM-NC have difficulties with dispersed datasets. TCM-SVM is so processing intensive that it suffers from large datasets. TCM-KP is only practicable to relatively noise-free data. In short, there are many restrictions on data qualities when applying them to real world data. The difficulties in essence lie in the nonconformity measure, which remains an unanswered question.

Taking above into account, we propose a new algorithm called CP-RF. Random forest classifier naturally leads to a dissimilarity measure between examples in a "strange" space rather than a Euclidean measure. After a RF is grown, since an individual tree is unpruned, the terminal nodes will contain only a small number of observations. Given a random forest of size *k*: *f *= {*T*_1_,..., *T*_*k*_} and two examples *x*_*i *_and *x*_*j*_, we propagate them down all the trees within *f*. Let *D*_*i *_= {*T*_1*i*_,... *T*_*ki*_} and *D*_*j *_= {*T*_1*j*_,... *T*_kj_} be tree node positions for *x*_*i *_and *x*_*j *_on all the *k *trees respectively, a random forest similarity between the two examples is defined as:

$$prox(i,j)=\frac{1}{k}\sum_{t=1}^{k}I(T_{ti},T_{tj})$$

where

$$I(T_{ti},T_{tj})=\{\begin{array}{ll} 1 & if\;T_{ti}=T_{tj}\\ 0 & else \end{array}$$

i.e., if instance *i *and *j *both land in the same terminal node, the proximity between *i *and *j *is increased by one, this forms a *N *× *N *matrix (⟦*prox*(*i*, *j*))⟧_*N *× *N*_, which is symmetric, positive definite and bounded above by 1, with the diagonal elements equal to 1, and *N *is the total number of cases [26].

Outliers are generally defined as cases that are removed from the main body of the data. In the framework of random forest, outliers are cases whose proximities to all other cases in the data are generally small. A useful revision is to define outliers relative to their class. Thus, an outlier in class *j *is a case whose proximities to all other class *j *cases are small. The raw outlier measure for case n in class *j *to the rest of the training data class *j *is defined as

(5) $$out_{raw}(i)=\frac{nsample}{\bar{p(i)}}$$

where *nsample *denotes the number of samples in class *j and *$\bar{p(i)}$ is the average proximity from case *i *to the rest of the training data within class *j*:

$$\bar{p(i)}=\sum_{j}{\left[prox(i,j)\right]}^{2}$$

The value of *out*_*raw*_(*i*) will be large if the average proximity is small. Within each class find the median of these raw measures $\bar{out_{raw}}$, and their absolute deviation *σ *from the median. The raw measure is scaled to arrive at the final outlier measure by the following:

(6) $$out(i)=\frac{out_{raw}(i)-\bar{out_{raw}}}{\sigma }$$

After a random forest is constructed, the proximity matrix of training dataset and a given test example remains the same regardless of changing the order of input data sequence, so random forest outlier measure can be used as a nonconformity measure.

In our method CP-RF, we define a new nonconformity measure *α*_*i *_= *out*(*i*), and then predict each test sample with Eq. (3). The detailed CP-RF algorithm is summarized in pseudo codes below.

Algorithm: CP-RF

**Input**: Training set *T *= {(*x*_1_, *y*_1_),..., (*x*_*l*_, *y*_1_)} and a new unlabeled example *x*_*l*+1_.

**Output**: The set of *p value*s $\left\{p_{l+1}^{1},...,p_{l+1}^{m}\right\}$ when *T *is an *m*-class dataset.

1. for *i *= 1 to *m *do

2. Assign label *i *to *x*_*l*+1_

3. Construct a RF classifier with training set *T*, put the test example *x*_*l*+1 _to the forest and output the sample proximity matrix (⟦*prox*(*i*, *j*))⟧_(*l*+1) × (*l*+1)_;

4. Compute nonconformity scores $\alpha_{1}^{i},...,\alpha_{l}^{i},\alpha_{l+1}^{i}$ of all examples using Eq.(6) ($\alpha_{l+1}^{i}$ is the nonconformity measure of *x*_*l*+1 _when assigned label *i*);

5. Compute the *p value *$p_{l+1}^{i}$ of *x*_*l*+1 _with Eq. (3).

6. End for

### Label conditional CP-RF algorithm

Given a significance level *ε *> 0 and the goal is to compute predictive regions, ideally consisting of just one label, containing the true label with probability 1 - *ε*. But in some situations our predictions are well-calibrated globally, but not within each class. In cost-sensitive learning problem, we must allow different significance levels to be specified for each possible classification of an object because the penalty of misclassification is not the same among all classes [27,28]. This problem can be viewed as a conditional inference. We extend our method to label conditional CP to address it, which can also be seen as one version of Mondrian CP (MCP) [3,29].

An important aspect of MCP is the method of calculating *p values*. For example, calculating the p-values in standard CP, the nonconformity score of a new example against the nonconformity scores of all examples observed up to that point are compared. In contrast, label conditional CPs compare the nonconformity score of a new example with the previously observed examples within each class. In detail, this method applies a function called Mondrian taxonomy to effectively partition the example space *Z *into rectangular groups. Given a division of the Cartesian product *N *× *Z *into categories: a function *k*: *N *× *Z *→ *K *maps each pair (*n*, *z*) (*z *is an example and *n *is the ordinal number of this example in the data sequence ⟦(*Z*)⟧ ↓ 1, *z *↓ 2,...) to its category; a label conditional nonconformity measure based on *k *is defined as:

*A*_*n*_: *K*^*n*-1 ^× ((*Z*^•^))^*K *^× *K *× *Z *→ R

The smoothed Mondrian conformal predictor (smoothed MCP) determined by the Mondrian nonconformity measure *A*_*n *_produces *p values *as:

(7) $$\begin{array}{c} \Gamma^{\varepsilon ,\tau_{n},k}(z_{1},...,z_{n-1},x_{n},\tau_{n},k)=\\ \left\{y\in Y:p_{y}=\frac{\left|\{i:k_{i}=k_{n}\&\alpha_{i}>\alpha_{n}\}|+\tau_{n}|\{i:k_{i}=k_{n}\&\alpha_{i}=\alpha_{n}\}\right|}{\left|\{i:k_{i}=k_{n}\}\right|}>\varepsilon \right\} \end{array}$$

with *α*_*i *_denotes a nonconformity score.

In label conditional CP-RF, *α*_*i *_= *out*(*i*) and compared with CP-RF, the small difference is computing *p values *with part of training examples, with Eq. (7). So we get higher computational efficiency. Limited by space, the detailed label conditional CP-RF algorithm is omitted here.

## Availability

The chronic gastritis dataset, the core source codes of CP-RF and label conditional CP-RF are available at  or 

## List of abbreviations used

CP: Conformal predictor; RF: Random forests; KNN: K nearest neighbour classifier; SVM: Support vector machine; KP: Kernel perceptron; NB: Naïve Bayes; NC: Nearest centroid; LDC: Linear discriminant classifier; KNC: Kernel nearest centroid; ANN: Artificial neural network.

## Competing interests

The authors declare that they have no competing interests.

## Authors' contributions

FY, HM and HZW conceived the study and research question. FY and HZW designed and implemented the algorithms, set up and performed the experiments and drafted the manuscript. HM, CDL and WWC contributed to the theoretical understanding and presentation of the problem.
